# Shared Experiences in Post‐Rehabilitation COPD Care Management: A Qualitative Study From Patient and Care Manager Perspectives

**DOI:** 10.1111/hex.70680

**Published:** 2026-05-05

**Authors:** Espérance Moine, Mathis Brusseau, François Alexandre, Sophie Gendrault, Nelly Heraud

**Affiliations:** ^1^ Scientific and Research Department Clariane Group Lodève France; ^2^ EuroMov Digital Health in Motion, Université de Montpellier, IMT Mines Alès Montpellier France; ^3^ Move in Med Baillargues France

**Keywords:** care manager, COPD patients, cross‐analysis, implementation, long term follow‐up, qualitative study, semi‐structured interviews

## Abstract

**Introduction:**

Achieving long‐term behavioural change in chronic disease management, particularly in chronic obstructive pulmonary disease (COPD), remains a significant challenge. Although maintenance programmes have been developed to extend the benefits of pulmonary rehabilitation, patient adherence is often comperomised by persistent symptoms, low motivation, and fragmented care. Research highlights the importance of therapeutic alliance, social support, and personalised follow‐up to encourage long‐term healthy behaviour. Care managers (CMs) may help facilitate these key elements by providing individualised support and coordination. The aim of this study is to identify key elements that support the sustainable implementation of the CM role in chronic care pathways, by exploring the shared experiences of COPD patients and CM involved in an 18‐month remote follow‐up post‐rehabilitation programme.

**Methods:**

A qualitative descriptive study was conducted using semi‐structured interviews with COPD patients and care managers who participated in the INSPIR'ACTION national experiment. This programme included an initial pulmonary rehabilitation phase followed by an 18‐month remote follow‐up. Interviews focused on the follow‐up phase and were analysed using inductive thematic analysis.

**Results:**

Data saturation was reached with a final sample of 9 patients and 7 CMs. Patients described CMs as supportive professionals who helped sustain motivation, adherence to healthy behaviours, and continuity of care. The relationship was perceived as trustful and personalised, even in a remote format. CMs expressed pride in their role but also reported organisational challenges, including lack of recognition and insufficient time allocation. Both groups emphasised the importance of relational continuity and individualised support.

**Conclusion:**

Patients and care managers described the CM as a key supportive figure offering personalised follow‐up perceived as helping promote therapeutic engagement and behavioural change. Relational continuity throughout the remote follow‐up was seen as fostering a trusting relationship that shaped participants' experience of the programme. By highlighting organisational elements that could influence implementation, our study may help inform future strategies to enhance the sustainable integration of care management in COPD care pathway.

**Patient or Public Contribution:**

COPD patients and CMs involved in the INSPIR'ACTION programme shared their experiences through interviews, helping to identify key factors for improving care manager support and long‐term follow‐up. Their input directly informed the study's findings and recommendations.

**Abbreviations**
CM: care manager
CNIL: National Committee for Computing and Liberties
COPD: chronic obstructive pulmonary disease
CRD: chronic respiratory disease
P: patient
SD: standard deviation
SRQR: Standards for Reporting Qualitative Research

## 1 Introduction

### 1.1 Background

Chronic diseases represent a major and growing burden for health systems worldwide [1, 2]. Among chronic conditions, chronic respiratory diseases (CRDs), and chronic obstructive pulmonary disease (COPD) in particular, represent a substantial burden. In 2021, nearly 82 million people were living with a CRD in the WHO European Region, and COPD accounted for around 80% of CRD‐related deaths [3, 4]. COPD is characterised by persistent respiratory symptoms and chronic airflow limitation, and is associated with progressive functional decline, dyspnoea, reduced exercise tolerance and recurrent exacerbations [5, 6]. Consequently, many patients experience limitations in daily activities and require coordinated, long‐term support [7, 8].

Pulmonary rehabilitation is a cornerstone of COPD management and is associated with improvements in symptoms, exercise capacity and quality of life [5, 9]. However, sustaining these gains after programme completion remains challenging: benefits often diminish within the first year, and maintaining long‐term health behaviours is difficult in the context of ongoing symptoms, fluctuating motivation and limited post‐programme support [6, 10]. This “post‐rehabilitation gap” reflects the challenge of transferring structured rehabilitation routines into everyday life, often compounded by fragmented follow‐up and limited care continuity [11, 12]. Behaviour change is a lengthy and complex process that typically requires long‐term support [13]. Habit formation typically takes 2 to 6 months, and up to a year for some behaviours, depending on individual characteristics and motivation [13, 14, 15, 16]. Therefore, structured follow‐up interventions are considered important to preserve therapeutic and behavioural gains after rehabilitation [17, 18].

In response, maintenance programmes are increasingly proposed to extend rehabilitation benefits, including remotely delivered follow‐up formats [19, 20]. In COPD, remote approaches using telephone, videoconferencing or digital platforms have been evaluated and may help sustain engagement when face‐to‐face follow‐up is logistically difficult [21, 22]. Across this literature, relational and motivational components such as therapeutic alliance, social support and personalised follow‐up, are frequently highlighted as key ingredients that may help patients remain engaged and sustain behaviour change over time [23, 24]. Nevertheless, there is still limited consensus on how to organise long‐term maintenance support after rehabilitation in a way that is both feasible and sustainable in routine care [5, 10]. In addition, interventions that include ongoing human support, grounded in empathy and shared decision‐making, tend to achieve better engagement and behavioural outcomes than purely self‐guided or AI‐only approaches [23, 24, 25].

Care management has been introduced in some chronic care pathways to strengthen continuity, support coordination and reinforce longer‐term behavioural goals [26]. Care managers (CMs) may provide structured follow‐up, facilitate multidisciplinary coordination and deliver tailored motivational support [27, 28]. They may also monitor adherence and motivation over time, with the aim of reducing disengagement and potential dropout from recommended behaviours or follow‐up [29]. Quantitative studies across chronic conditions have reported improvements in outcomes such as quality of life, treatment adherence and healthcare utilisation in some contexts, although findings appear heterogeneous and dependent on programme design and setting [30, 31, 32]. Importantly, the CM role remains variably defined and unevenly established across chronic disease pathways and health systems; more structured CM models have been described mainly in primary care settings and have been extensively documented in mental health programmes (*e.g*., depression) [33, 34].

In COPD, qualitative evidence remains scarce regarding care management in long‐term post‐rehabilitation pathways, particularly when follow‐up is delivered remotely over extended periods. More broadly, existing qualitative studies on care management have largely focused on primary care with mid‐term follow‐up (often 3–6 months) and rarely cross‐reference patient and CM perspectives to capture shared experiences and implementation conditions [35, 36, 37]. In particular, it is unclear how care managers (CMs) operationalise post‐rehabilitation support over time (*e.g.*, sustaining engagement, coordinating care, tailoring follow‐up, and supporting behaviour change), and which organisational and relational conditions may enable or constrain this work [28, 33]. Cross‐referenced accounts from patients and CMs are therefore needed to clarify what care management entails in practice in COPD follow‐up and to identify contextual factors relevant to its integration within COPD care pathways.

### 1.2 Objective

This research aims to develop a comprehensive understanding of the long‐term role of care managers by exploring the shared experiences of patients and professionals, and identifying key elements to support the sustainable implementation of this role within an individualised care pathway for chronic respiratory disease, integrating remote follow‐up after pulmonary rehabilitation.

## 2 Methods

### 2.1 Design

This qualitative descriptive interview study was conducted prospectively to explore the experiences of care managers and patients involved in an eighteen‐month remotely delivered follow‐up experiment, piloted by pulmonary rehabilitation centres. Semi‐structured interviews were conducted during and at the end of the experiment to describe participants' perspectives on the care manager role and the follow‐up process. Qualitative interviews capture individuals' experiences, including emotions, opinions and thoughts [38].

### 2.2 Ethical Considerations

The study was conducted in accordance with French data protection regulations for non‐biomedical research involving human participants (National Committee for Computing and Liberties (CNIL), reference methodology no.004, Deliberation no.2018‐155 of 3 May 2018). The protocol and statistical analysis plan have been pre‐registered on clinicaltrials.gov (NCT06448559) and on the French Health Data Hub platform (no.18686153). Patients and professionals who had been part of the experiment were contacted for participation in this study and were given written information explaining the objectives of the project. All participants provided verbal consent directly before the interview was conducted and audio recorded. Participants were informed that their participation was voluntary and that they could withdraw from the study at any time.

### 2.3 Participants and Recruitment

Participants were recruited during the follow‐up phase of the INSPIR'ACTION experiment, conducted across ten pulmonary rehabilitation centres in France and funded by the Ministry of Health. This experiment aimed to improve access to care, support personalised health and lifestyle changes, and ensure coordinated follow‐up to reduce avoidable complications for patients with COPD through a two‐phase programme: an intensive phase (lasting 2 to 8 weeks), and a remote support phase of 18 months. The initial phase included various formats and durations of multidisciplinary pulmonary rehabilitation, tailored to patient needs: a 2‐ or 3‐week inpatient rehabilitation programme, an 18‐session outpatient rehabilitation programme, or an 18‐session telerehabilitation programme. Following this initial rehabilitation phase, patients received remote support from home over 18 months, coordinated by a CM. This support consisted of two follow‐up contacts per month for the first 6 months and one follow‐up contact per month for the remaining 12 months, conducted by telephone or videoconference. The 18‐month duration was predefined by the INSPIR'ACTION programme design. This timeframe was selected to extend beyond the first year after rehabilitation (when gains may decline) and to allow a longer consolidation period for sustaining behaviour change. For the present qualitative study, this extended follow‐up provided an opportunity to examine organisational and relational dynamics of care management over time. CMs were healthcare professionals employed within each participating pulmonary rehabilitation centre, with dedicated time allocation varying across sites. Patients were typically assigned to the CM designated for their rehabilitation centre, which structurally supported relational continuity throughout the 18‐month follow‐up period.

The study targeted both COPD patients and care managers. Patient inclusion criteria were: i) participation in the INSPIR'ACTION programme, ii) at least 6 months of follow‐up with the CM, and iii) completion of a minimum of 30% of scheduled follow‐up contacts with the CM over the 18‐month follow‐up. Exclusion criteria included being a protected/vulnerable adult, or having completed follow‐up more than 4 months ago. Eligible patients were identified across the eight participating institutions. Two patients meeting the criteria were randomly selected from the active patient list of each CM. In the first step, the corresponding CMs were informed of the selection and asked to contact the random patients. They provided study information and patients who agreed to participate gave their verbal consent to be contacted by a study investigator. In the second step, the investigator reached out to the patient to conduct the interview, with participants reaffirming their consent at the beginning of the interview.

CM inclusion criteria were: i) at least 6 months of experience in the CM role, and ii) having supported more than ten patients within the INSPIR'ACTION programme. Eligible CMs were identified across the participating institutions. Investigators contacted those meeting the criteria to provide study information and scheduled interviews with those who agreed to participate. At the beginning of each interview, participants were asked to reaffirm their consent.

### 2.4 Procedures and Data Collection

Interviews were conducted via video calls, scheduled according to participant's availability, between August 2024 and February 2025. All interviews were conducted remotely in French using Microsoft Teams.

A semi‐structured interview guide was designed to assist investigators during the interviews. A detailed list of interview questions for both patients and CMs is provided in Supporting Information: Material S1. The first 5 min of each session were dedicated to introducing the interview, explaining confidentiality terms, and obtaining verbal consent. Recording was initiated using the integrated Teams feature, and each interview lasted approximately 20 min. Audio recordings were transcribed using Microsoft Word's transcription tool. To ensure transcription accuracy, the audio was replayed while carefully proofreading the text.

For patient participants, sociodemographic data were collected, including sex, age, employment status, modality of initial rehabilitation care, duration within the care pathway, and the percentage of meetings completed. For CMs, collected data included sex, age range, initial profession, length of service at the care centre, and duration in the care manager role.

### 2.5 Researcher Characteristics and Reflexivity

Semi‐structured interviews were conducted by two independent interviewers: one interviewed care managers and the other interviewed patients. Neither interviewer was involved in the design or delivery of the INSPIR'ACTION programme, nor in the conception of this qualitative study, and neither had any prior contact or specific relationship with the CMs and patients interviewed. To enhance consistency and minimise interviewer influence, both interviewers followed the semi‐structured interview guide developed by the study team.

Data were analysed inductively by two researchers (EM and MB). Codes and themes were subsequently compared, discussed, and refined to consensus during analytic meetings with two additional researchers (FA and NH) who had not participated in the coding, providing an external perspective on interpretation. Finally, findings were presented to a research committee to further review and validate the interpretive conclusions.

### 2.6 Data Analysis

Interviews continued until data saturation was reached. The following saturation process, based on the method published by Guest et al., was used for both care managers and patients [39]. When assessing saturation, incoming information is weighed against the information already obtained. This saturation analysis method includes three primary elements which are the base size, the run length, and the new information threshold. In this study, the base size was set at 4 interviews and corresponds to the amount of information identified in a dataset to be used as the denominator. The run length, or the number of interviews within which we search for new information, was set at 3. The newly identified codes are then used as the numerator. The new information threshold, used to indicate saturation and corresponding to the acceptable level of new information between run length and base size, has been set to < 5%. The saturation analysis results are available in the Supporting Information: Material S2.

The inductive thematic analysis was then performed, according to Braun & Clarkes' approach and is divided into six phases, during which researchers may have to go back and forth [40]. The entire process was conducted by two researchers (EM and MB). Phase 1, familiarisation: Transcripts of interviews were read multiple times to become familiar with the data. Phase 2, generating initial codes: Each interview was systematically coded, searching for features that were relevant to the aim, using the software QualCoder version 3.5. Phase 3, building themes and categories: Codes were examined to identify fragments of meaning and generate initial themes and categories. Phases 4 and 5, revising and defining/naming themes and categories: Themes were checked against the coded data to ensure they reflected the whole dataset. During this process, themes were reviewed and further developed, informative names were constructed, and final themes and categories were defined. Phase 6, producing the report: The final themes and subthemes and their meanings are presented in the results of this report, illustrated by quotes. The final coding and the final themes and categories were named by consensus. The analysis was contextualised in relation to existing research.

To evaluate the quality and rigour of this qualitative research, we applied Braun et al.'s 15‐point checklist of criteria for good thematic analysis [40]. The completed checklist is available in the Supporting Information: Material S5. This study followed the principles outlined in the Standards for Reporting Qualitative Research (SRQR), as detailed in Supporting Information: Material S6 [41].

## 3 Results

### 3.1 Characteristics of Participants

#### 3.1.1 Patients With COPD

From October 2024 to February 2025, 16 patients meeting inclusion criteria were drawn at random. 9 of them agreed to take part in the semi‐structured interview and were necessary to reach saturation. Their characteristics are presented in Table 1. The interviewed patients were recruited from six different rehabilitation centres. Each patient was consistently followed by the same CM throughout the programme, although interviewed patients may have been supported by different CMs.

**Table 1:** Baseline characteristics for all patients.

Variables		*N* = 9
Sex ratio (male/female)		4/5
Age (years)		65.6 ± 10.5
Employment status, *n* (%)	Retired	6 (66%)
	Unemployed	1 (11%)
	Missing information	2 (22%)
Situation at the time of interview, *n* (%)	In follow‐up	7 (77%)
	Follow‐up ended	2 (23%)
Follow‐up session with CM completed (%)		66 ± 17

*Note:* Data are presented as means ± SD or *n* (%).

#### 3.1.2 Care Managers

From October 2024 to February 2025, 13 eligible CMs were identified. Nine of them agreed to take part in the semi‐structured interview, but only the first seven interviews were analysed as saturation had been reached. Saturation was reached after seven interviews. The characteristics of the CMs are presented in Table 2. The professionals came from diverse backgrounds (i.e., nursing, kinesiology, physiology) and had generally been working in the CM role for over 1 year.

**Table 2:** Baseline characteristics for all care managers.

Variables		*N* = 7
Sex ratio (male/female)		2/5
Age range, *n* (%)	31–40 years	3 (43%)
	41–50 years	4 (57%)
Initial job degree, *n* (%)	Nurse	3 (43%)
	Physical activity teacher (kinesiologist)	3 (43%)
	Physiologist	1 (14%)
Length of service in the care centre, *n* (%)	2–5 years	1 (14%)
	5–10 years	3 (43%)
	> 10 years	3 (43%)
Time in the role of CM, *n* (%)	6–9 months	1 (14%)
	13–18 months	3 (43%)
	19–24 months	3 (43%)

*Note:* Data are presented as *n* (%).

Abbreviation: CM, care manager.

### 3.2 Analysis of the Interviews

#### 3.2.1 Thematic Analysis

##### 3.2.1.1 Patient Interviews

**Figure 1:**
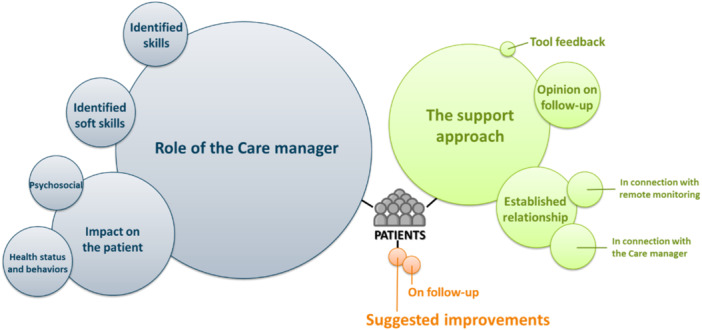
Thematic map of patient interviews.

The thematic analysis of the patient interviews identified 51 codes. They were grouped into three main categories, each of which was subdivided into themes and sub‐themes where appropriate, as shown in the thematic map in Figure 1. A full list of all the codes identified, along with their occurrence, is available in the Supporting Information: Material S3.

The first category identified relates to the role of the care manager. Patients identified the professional and the soft skills of their care managers. They also described the impact of the care manager on their psychosocial, health and behavioural status. Among the most frequent interview codes related to identified skills and soft skills, they reported that their care managers monitored their health, provided information and support, and acted as a counsellor. Patients endorsed their care manager's professionalism and listening skills. Regarding the impact of the care manager on the patients, all participants described enhanced motivation, mainly as an extrinsic reinforcement linked to regular contact that encouraged them to maintain efforts and reduced the risk of disengagement over time. A majority also reported shifts in perceptions, which primarily concerned how they viewed the management of their disease and their own ability to act (self‐efficacy) in everyday life. These changes were described as translating into greater engagement in health behaviours, including increased physical activity and treatment adherence. The second category identified is related to the support approach. All patients expressed satisfaction with the follow‐up and its remote modality. In terms of the relationship established, they emphasised the benefits of contact with their care manager and the fact that they felt positively controlled or pressured to follow the programme. The third category is related to suggested improvements to make the care manager's support even more in line with the patient's expectations. The suggested improvements were associated with the follow‐up and questionnaire provided, but their frequency is minimal and the most reported code in this category is the absence of suggested improvement.

##### 3.2.1.2 Care Manager Interviews

**Figure 2:**
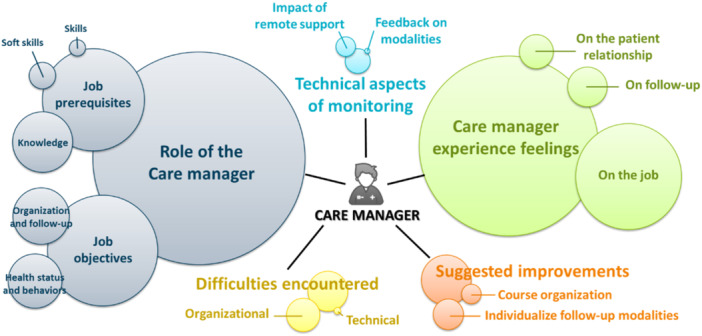
Thematic map of care manager interviews.

The thematic analysis of the care manager interviews identified 89 codes. They were grouped into five main categories, each of which was subdivided into themes and sub‐themes where appropriate, as shown in the thematic map in Figure 2. A full list of all the codes identified, along with their occurrence, is available in the Supporting Information: Material S4.

The first category identified relates to the role of the care manager. Among the most prevalent codes, they report the need to work as part of a multidisciplinary team and define themselves as a relay or link both within and outside the structure. The objectives they report are to provide support and advice, and to act as a referral coordinator for patients. The second category corresponds to the feelings experienced by care managers in relation to patient relationships, follow‐up and their job. Care managers emphasised the importance of this profession, the strong personal relationships they build with patients, and their sense of pride and satisfaction in this approach. However, they also mentioned feelings of loneliness and helplessness, and the time‐consuming nature of the role. The third category represents the suggested improvements to the organisation of the course and the individualisation of follow‐up modalities, with a particular focus on the necessity to personalise the frequency and duration of follow‐ups according to the needs of each patient. The fourth category refers to the organisational and technical difficulties encountered by care managers, such as a lack of recognition and insufficient time dedicated to this mission. Finally, the fifth category is dedicated to the technical aspects of monitoring, focusing on the impact of remote support and feedback on modalities. Care managers reported that follow‐up by telephone or videoconference was workable over time, preserved the quality of the patient relationship, and was not a barrier to delivering monitoring and support. Videoconferencing was even perceived as fostering proximity and better integration into patients' everyday lives outside rehabilitation.

#### 3.2.2 Crossed Analyses

##### 3.2.2.1 Missions of the Care Manager

The care manager's role was clearly defined by both patients and professionals. It appears that their role is to coordinate patient care and monitor health status in conjunction with other professionals. “*his/her role is to call me regularly to find out about my state of health*” – P4. “*This role acts as a bridge between patients, caregivers, medical and paramedical staff, external structures, and external stakeholders*” – CM2. Care managers also illustrated how coordination occurred in practice: during follow‐up contacts, they identified emerging needs with the patient and could mobilise other professionals within the centre (*e.g.*, psychologist or dietitian), while relaying relevant information to the broader clinical team. “*If an individual consultation is needed, we are often the ones who contact the health professionals, for example when there is a specific need to work with the psychologist*” – CM7. “*I bring up what is going on with patients and their follow‐up with the referring pulmonologists here and with the nurses*” – CM2. An important part of this job involves providing information and advice to patients according to their needs. “*He/she also sent me exercises that I can do at home with photos*” – P7. “*be there to answer their questions as needed*” – CM1. Patients praised their care managers for providing support, active listening and professionalism. “*you can feel the professionalism*” – P1. “*a very competent person*” – P8. “*They're very good listeners*” – P6.

##### 3.2.2.2 A Positive Impact on Adherence to Healthy Behaviours

A key element of this support is maintaining the patients' motivation towards their therapeutic objectives through regular contact with the care manager. “*I think that if I didn't have these contacts every month, maybe I would have given up*.” – P2. “*This little external stimulus (motivation) is still extremely important.*” – CM4. This support also promotes the adoption of healthy behaviours and improves patient autonomy. “*I do more physical activity. I ride my bike more at home*” – P7. “*It allowed me to reflect a little on the way I used to live*” – P2. “*They have incorporated this (healthy behaviour) into their lifestyle, which is what we were aiming for*.” – CM4.

##### 3.2.2.3 Patient‐Care Manager Relationship

One major finding from the interviews was the importance of the relationship established between care managers and their patients. This relationship was described as being very strong and based on honesty and trust, as well as being more personal than the traditional caregiver‐patient relationship. “*we are in a relationship of communication and exchange*” – P3. “*As time goes by, we get to know the whole family*” – CM1. Patients also reported feeling under a certain amount of pressure or supervision due to regular calls from care managers. This motivated them to do the prescribed exercises and stick to their goals, as they had to report back. “*to know that she's going to call and ask me questions about what I'm doing, it's a real incentive to do it*” – P7. At the same time, the CS reported that this long‐term, privileged relationship enabled them to gain a better understanding of their patients and their living environments. This improved their understanding of the issues that patients face in their daily life. “*This really helps us understand the difficulties they (patients) may encounter on a daily basis*.” – CM5. “*we know our patient better, we know his way of thinking better*” – CM7.

##### 3.2.2.4 Monitoring and Tools

The specific relation between the patient and the CM is maintained by remote tools, such as telephone calls or videoconference. CMs reported adapting the modality to patients' needs and digital ease, using telephone follow‐up when videoconferencing was not feasible. Patients and CMs are satisfied with this remote modality. “*Videoconference and telephone calls are good, and the two can complement each other*.” – P5. Patients and CMs reported that remote modality does not alter the relationship that is sometimes initiated in the face‐to‐face setting during the rehabilitation phase. “*It's like being face‐to‐face, but you don't have to leave your home*” – P6. However, CMs noted that patients who had not experienced telerehabilitation could be more hesitant about connecting for videoconferencing, making telephone contacts particularly useful to support the transition to remote follow‐up. “*People who didn't do telerehabilitation… they are more reluctant*” – CM4. Telerehabilitation and remote follow‐up were also described as expanding access to patients who would not have engaged in inpatient rehabilitation. “*It allows us to reach other people who would potentially never have come for hospitalisation*” – CM4. In addition, lack of prior face‐to‐face contact did not necessarily prevent a good interaction. “*I didn't know him, but it didn't prevent the consultation from going well*” – P7. Overall, patients and CMs were satisfied with the monitoring. “*It's a good surprise for everybody, for patients and for my colleagues*” – CM5.

#### 3.2.3 Insights Into CMs' Lived Experience

##### 3.2.3.1 Feelings on Their New Role

CMs reported feeling lonely in their role as care managers, and said that facing certain situations with their patients could make them feel helpless or powerless. “*We do feel a bit lonely when we're a care manager, though*.” – CM7. “*Sometimes I feel a little bit helpless*.” – CM4. “*Sometimes it's difficult to refer the patient*.” – CM6.

On a positive note, CMs reported feeling proud of their role. They emphasised the development of their skills throughout the process. Furthermore, they are convinced of the importance of the care manager role and would highly recommend it. “*it's extremely rewarding*” – CM3. “*I think this is something that is sorely lacking (the care manager) in healthcare facilities that treat chronic patients today*.” – CM5. “*I would recommend it (the role of care manager) because it's actually very interesting*.” – CM1.

##### 3.2.3.2 Difficulties Encountered

The major difficulties reported by CMs relate to the organisation around their missions. Technical difficulties were rarely mentioned, accounting for only 1% of all feedback. Some CMs reported an insufficient amount of time dedicated to accomplishing their missions, as most held part‐time positions to manage their mission. “*It's frustrating to think that I'm always running out of time*.” – CM4. “*Perhaps there wasn't enough time to manage the missions*.” – CM7. The missions were sometimes out of step with the medical teams, as the CMs support patients who were not currently undergoing rehabilitation. This can sometimes lead to a lack of recognition. “*We are not on the usual path; we're walking alongside it.*” – CM4.

##### 3.2.3.3 Areas for Improvement

CMs mentioned areas for improvements, particularly in terms of personalised monitoring. They pointed out the difference in needs for patients, in terms of autonomy, which requires individualised intervention content. “*Some patients need more autonomy, and others need more support*” – CM3. The CMs also mentioned the need to improve frequency and duration by individualising it to each patient. “*A year and half may be a long time for monitoring*” – CM6. “*Maybe more flexibility in terms of support frequency*” – CM3.

On the contrary, patients did not suggest areas for improvement or future perspectives. Instead, they emphasised that the support provided was already very satisfactory. “*I don't really know what could be better*” – P7.

## 4 Discussion

This qualitative study aimed to deepen understanding of the long‐term role of Care Managers (CMs) within an experimental individualised care pathway that includes an 18‐month follow‐up after a rehabilitation among COPD patients. Through semi‐structured interviews with patients and CMs, we explored their shared experiences and identified key challenges and potential areas for improvement to facilitate and support the sustainable implementation of this role. The findings indicate that the CM's role is well understood and positively perceived. Both patients and CMs reported that CMs contributed to improved communication among healthcare professionals, thereby enhancing care continuity. The follow‐up also boosted patients' motivation and engagement with therapeutic goals and healthy behaviours. A key insight is the strong, trust‐based relationship developed between CMs and patients, often extending beyond a formal professional interaction. The remote format was well accepted by both groups. However, CMs reported that their role remains insufficiently recognised within and outside their institutions.

In light of these findings, it is particularly noteworthy that patients clearly identified the CM's role as helping them maintain health behaviours and providing regular monitoring. This monitoring was not perceived as intrusive or controlling, but rather as a form of support that helped them stay engaged with their therapeutic goals. The CM was seen as a reliable and available resource, capable of answering questions, providing tailored advice, and adapting to individual needs. These perceptions echo previous studies showing that patients value the CM's presence as a facilitator of continuity and coordination in care [35, 42]. Importantly, the CM's role was not reduced to technical monitoring or data collection. Patients emphasised the human dimension of the support, which they distinguished from digital or automated follow‐up systems. Unlike most primary care studies with follow‐ups limited to 6 months, our findings highlight the added value of an 18‐month post‐rehabilitation follow‐up, as reported by patients and CMs. This extended support occurs during a critical period for sustaining behavioural change, consistent with behavioural science literature emphasising the importance of social support and therapeutic alliance in long‐term adherence [43, 44]. These results clarify how the CM can play a central role in the long‐term support of patients, where sustaining engagement and the maintenance of health behaviours are essential, thereby reinforcing its potential as a scalable solution for COPD care pathways.

The relationship between patients and CMs emerged as a central component of the intervention's success. Patients described a bond based on trust, honesty, and mutual understanding, which extended beyond the typical caregiver‐patient dynamic. This relational depth enabled CMs to better understand patients' environments, challenges, and motivations, and to tailor their support accordingly. From an implementation perspective in the post‐rehabilitation setting, these findings underscore the importance of relational continuity. Previous studies have shown that such stable relationships contribute to improved adherence, reduced psychological distress, and enhanced patient satisfaction [33, 34, 45]. In our study, being followed by the same CM throughout the programme helped the patients to feel understood and supported. This continuity was largely supported by the programme organisation, in which CMs were embedded within each rehabilitation centre and patients were generally followed by the CM designated for their centre. These findings suggest that continuity could foster trust and help mitigate the fragmentation often associated with interchangeable personnel, making it a potentially key element for successful implementation.

The interviews also revealed the importance of motivational support provided by CMs. Patients reported that regular contact helped them maintain their engagement with therapeutic goals and avoid disengagement over time. In practice, this support was enacted through structured, scheduled follow‐up contacts in which CMs helped patients define individual goals, checked in on health status and routinely reviewed progress toward agreed therapeutic objectives, providing feedback and adjusting plans when needed. This motivational reinforcement was not limited to extrinsic reminders, but also contributed to deeper forms of regulation, such as internalised motivation and self‐reflection. These findings resonate with the Behaviour Change Wheel framework [43], which identifies capability, opportunity, and motivation as key conditions for behaviour change. CMs appeared to influence all three: by enhancing psychological capability through education, reassurance and answering questions as needed, by creating opportunities through personalised guidance and coordination with other professionals when additional support was required, and by sustaining motivation through relational support and regular accountability. These practices reflect several Behaviour Change Techniques described by Michie et al., including goal setting, feedback, problem‐solving, and action planning, which CMs mobilised flexibly depending on each patient's needs [46]. Repeated contacts also enabled problem‐focused discussions about everyday barriers, supporting tailoring to patients' living context over time. Patients' feedback suggests that the CM's involvement helped them move from externally driven behaviours to more autonomous forms of regulation, consistent with self‐determination theory [47, 48]. Taken together, our findings suggest that patients perceive motivational support as a core component, and that the CM may be particularly well positioned to provide it, thereby facilitating sustained long‐term adherence.

These relational and motivational dynamics were preserved even in the context of remote follow‐up, which was initially considered a challenge. Both patients and professionals reported high levels of satisfaction with videoconferencing and telephone modalities. These formats did not compromise the quality of the relationship or the effectiveness of the support provided. This finding is particularly relevant for large‐scale implementation of post‐rehabilitation follow‐up, where in‐person monitoring may be logistically and financially unfeasible. The success of remote care management confirms that distance does not compromise the essential factors contributing to its effectiveness, such as trust, availability, and social support. This is consistent with previous research showing that remote interventions can improve physical activity, self‐efficacy, and quality of life in COPD patients [21, 22, 49]. Our results further confirm that remote care management is not a barrier, even over extended periods such as 18 months, for which no prior data were available.

Beyond the overall positive experience, the study also revealed significant challenges faced by CMs, particularly related to time constraints and lack of recognition. The role was described as demanding, requiring multitasking, coordination across disciplines, and constant availability to respond to patient needs or alerts. These constraints reflect the inherent complexity of the CM role, which involves supporting patients at different stages of their care pathway and adapting to evolving needs. The lack of institutional recognition was also highlighted, particularly in contexts where the CM's missions were not fully integrated into existing care structures. This sense of isolation is consistent with findings from other studies on emerging roles in chronic care, where unclear role definitions and limited visibility can hinder collaboration and effectiveness [28, 50, 51]. Addressing these challenges is essential to ensure the sustainability of the CM role. Failure to do so could lead to professional disengagement and turnover, which would compromise the relational continuity that patients value and that appears essential to the programme's success [52]. As suggested in the literature [53, 54, 55], organisational improvements such as increasing working hours, improving the preparation of follow‐up sessions and clarifying role responsibilities could enhance the CM's effectiveness. These constraints also have implications for feasibility and scalability in settings where CMs are not routinely embedded. In our context, the CM role was implemented within a structured programme with clearly defined missions, dedicated time allocation, and a programme‑defined schedule of remote follow‑up contacts designed to align with real‑world sustainability. Importantly, both patients and CMs perceived the frequency and format of contacts as acceptable and manageable, supporting the potential operational viability of this model. From an implementation‐science perspective, and in line with the Consolidated Framework for Implementation Research, these findings underscore the importance of organisational resources and role clarity, while also highlighting that policies and funding mechanisms will ultimately determine whether the necessary workforce time and support can be sustained at scale [56, 57].

At the same time, the interviews highlighted strong professional buy‐in and perceived value of the CM role. CMs expressed a strong sense of pride in their role and a deep conviction of its importance. Many described the experience as personally enriching and professionally meaningful. This sense of purpose, combined with the positive feedback from patients, reinforces the relevance of the CM role in chronic care pathways. It also suggests that, with appropriate institutional support and recognition, the profession could attract and retain committed individuals capable of delivering high‐quality, person‐centred care [58]. The enthusiasm and engagement expressed by CMs in this study could also indicate their willingness to remain involved in continuity of care and to contribute to improving long‐term patient management, offering a promising foundation for future development and scaling of care management models.

From a methodological point of view, this study presents several strengths, notably the use of neutral interviewers who were not involved in the INSPIR'ACTION experiment or the study's design, thereby minimising potential bias. Furthermore, data saturation analysis was employed to ensure the adequacy and completeness of the collected data. Thematic analysis was then conducted following Braun and Clarke's framework, including the application of their 15‐point checklist to assess the methodological rigour of the study. However, some limitations should be acknowledged. The recruitment of patients and care managers was context‐specific, limited to participants in the INSPIR'ACTION experiment within a post‐rehabilitation setting, which may affect the representativeness and generalisability of the findings. However, the convergence of the themes identified in our study and the content provided by the participants towards previous findings in the literature suggests that common principles underpin effective care management across care pathways, despite contextual differences [33, 35, 36, 37]. In addition to these considerations, we initially intended to include interviews with patients' caregivers to enrich the perspectives on the follow‐up process and provide a more external viewpoint on the programme. However, this component had to be abandoned, as none of the randomly selected patients had identifiable caregivers. Consequently, the interviews were limited to patients and care managers, potentially omitting a third, complementary perspective on the proposed long‐term follow‐up. Additionally, we included only patients who had been sufficiently exposed to the CM follow‐up to provide meaningful insights into the long‐term support process. In the broader INSPIR'ACTION experiment, however, only 10% of patients either discontinued participation or completed fewer than 30% of the scheduled follow‐up contacts, indicating overall good retention and follow‐up completion. This suggests that the predominantly positive feedback regarding the follow‐up modality is broadly representative of the overall population involved.

## 5 Conclusions

This qualitative study highlights the care manage as a pivotal figure in post‐rehabilitation COPD care pathways. Patients and care managers consistently described the CM as a supportive and accessible professional, with whom a trust‑based relationship was co‑constructed over time, and who provided personalised motivational support perceived as fostering therapeutic engagement and the maintenance of recommended health behaviours, even remotely. This care management approach, perceived by participants as providing ongoing relational support, may help sustain engagement with recommended behaviours and improve patient experience. However, organisational barriers such as time constraints and insufficient recognition could compromise sustainability and should raise concern about potential risk of professional disengagement. Future research should assess its generalisation beyond post‐rehabilitation contexts, evaluate cost‐effectiveness, and identify institutional strategies to support its implementation.

## Author Contributions

**Espérance Moine:** conceptualisation, methodology, writing – original draft, writing – review and editing, data curation, formal analysis, supervision. **Mathis Brusseau:** investigation, writing – original draft, writing – review and editing, data curation, formal analysis. **François Alexandre:** conceptualisation, writing – review and editing, methodology, validation. **Sophie Gendrault:** writing – review and editing, supervision. **Nelly Heraud:** conceptualisation, methodology, writing – review and editing, validation, supervision.

## Funding

The authors have nothing to report.

## Conflicts of Interest

The authors declare no conflicts of interest.

## Supporting information

Supporting File

## Data Availability

The data that support the findings of this study are available from the corresponding author upon reasonable request. Full interview transcripts and videoconference recordings are not publicly available due to data protection regulations. However, metadata, including the codebook and anonymized extracts that support the findings are available upon reasonable request by contacting the corresponding author via email.
