# Supplementary material for: Shared Experiences in Post‐Rehabilitation COPD Care Management: A Qualitative Study From Patient and Care Manager Perspectives
Source: Health Expect. 2026 May 5;29(3):e70680. doi: 10.1111/hex.70680 (PMC13145347; doi:10.1111/hex.70680)
Supplement: Supplementary file 1 — Supporting File [file HEX-29-e70680-s001.docx]

**Title**: **Shared Experiences in post-rehabilitation COPD Care Management: A Qualitative Study from Patient and Care Manager Perspectives.**

**RUNNING TITLE:** COPD Care Management: Qualitative Views

**SUPPLEMENTARY MATERIALS**

- S1: Interview questions for patients and care managers
- S2: Saturation analyses for patients and care managers
- S3: Table of identified codes, themes and categories for patient interviews
- S4: Table of identified codes, themes and categories for professional interviews
- S5: 15-point checklist of criteria for good thematic analysis
- S6: Standards for Reporting Qualitative Research (SRQR) checklist

**S1: Interview questions for COPD patients and care managers**

**PATIENTS**

Q1.1: You have taken part in an experiment which has led to you being monitored by a care manager. Describe/define the role of the care manager. In other words, for you, the care manager is someone who...

Q1.2: What do you think of the exchanges and the relationship you have established with the care manager?

Q1.3: How has the day-to-day management of your disease changed since you started communicating with the care manager?

Q2.1: What is your opinion of the remote exchanges (telephone interviews, videoconferences, e-mails, SMS, etc.) set up with the care manager? (frequency, duration, etc.)

Q2.2: How have remote exchanges (telephone interviews, videoconferences, e-mails, SMS, etc.) with the care manager changed the relationship between caregiver and receiver?

Q3.1: What improvements would you suggest making the care manager's support even more in line with your expectations?

**PROFESSIONALS**

Q1.1: Describe/define your role as a patient care manager. In other words, for you, the care manager is someone who...

Q1.2: How do you consider your position in relation to the other actors in the institution?

Q1.3: How would you (un)advise a colleague to become a care manager?

Q2.1: What challenges (technical, organizational, professional...) did you face to fulfil the role of care manager?

Q2.2: How have remote exchanges (telephone interviews, videoconferences, e-mails, SMS, etc.) with patients changed the relationship between caregiver and receiver?

Q3.1: How would you rate the service provided by the care manager at your facility?

Q3.2: What improvements would you suggest ensuring that care manager support meets the expectations of patients, facilities and the healthcare system?

**•S2: Saturation analyses for COPD patients and care managers**

For COPD patients, saturation was reached after nine interviews. The number of new codes per interview is shown in Table 1. The base size was 47 codes. Three runs were necessary to reach saturation, with 1 new code identified in the interviews of patients n°7 to n°9, resulting in a new information threshold of 2.13%.

**Table S2: Number of codes identified in interviews with Patients and Care managers**

| **COPD Patient ID** | **1** | **2** | **3** | **4** | **5** | **6** | **7** | **8** | **9** |
| --- | --- | --- | --- | --- | --- | --- | --- | --- | --- |
| New codes (n) | 35 | 6 | 2 | 4 | 1 | 2 | 0 | 1 | 0 |
| **Care manager ID** | **1** | **2** | **3** | **4** | **5** | **6** | **7** |  |  |
| New codes (n) | 50 | 19 | 9 | 7 | 0 | 1 | 3 |  |  |

For the care managers, saturation was reached after seven interviews. The number of new codes per interview is shown in Table 1. The base size was 85 codes. One run was required to reach saturation, with 4 new codes identified in the interviews of care managers n°5 to n°7, resulting in a new information threshold of 4.71%.

**S3: Table of identified codes, themes and categories for patient interviews**

| CATEGORIES | THEME | SUB-THEME | CODES and headcounts | | | |
| --- | --- | --- | --- | --- | --- | --- |
| **Role of the care manager** | Identified skills |  | - **Health monitoring (general)** - **Inform (provide support)** - **Advisor (opinion)** | **30**  **16**  **15** | - Support - Supporting smoking cessation - Relay, link with medical team | 13  8  2 |
|  | Identified soft skills |  | - **Professionalism** - **Listening** - Reactivity/Availability - Human/Caring | **18**  **15**  12  10 | - Adaptability/Flexibility - Positive/Pleasant - Reassuring - Building confidence | 9  9  9  7 |
|  | Impact on the patient | Psychosocial | - **Motivation towards therapeutic objectives** - **Changes in perceptions** - Emotion management | **31**  **23**  6 | - Patient reassured - Return to work | 5  4 |
|  |  | Health status and behaviours | - **Practice a physical activity** - **Treatment compliance** - Behaviour change | **24**  **22**  14 | - Defining a rehabilitation objective - Symptoms improvement - See the benefits | 13  8  7 |
| **The support approach** | Established relationship | In connection with remote monitoring | - **Satisfaction with remote modality** - Relationship unchanged by remote access - Prefer face-to-face if no distance | **18**  10  5 | - Prefer remote to face-to-face - Face-to-face is indispensable before remote - No need for face-to-face | 5  5  2 |
|  |  | In connection with the Care manager | - **Pressure/Control** - Healthy / honest relationship - Non-medical exchanges | **15**  14  12 | - Landmark - Attachment - Limits loneliness | 8  5  4 |
|  | Opinion on follow-up |  | - **Follow-up satisfaction** - **Beneficial contact** - Adapted follow-up timeframe | **37**  **19**  14 | - An enriching approach - Concern about the end of the follow-up | 8  7 |
|  | Tool feedback |  | - Face-to-face meeting with Care manager - IT problems - Questionnaire - Follow-up support | 9  4  3 | - Preference for videoconference - Visio not mandatory | 2  1 |
| **Suggested improvements** | On follow-up |  | - No suggestions for improvement - Improve questionnaire content | 10  6 | - Diversify questionnaires - Face-to-face assessment/monitoring | 4  4 |

**S4: Table of identified codes, themes and categories for professional interviews**

| CATEGORIES | THEME | SUB-THEME | CODES and headcounts | | | |
| --- | --- | --- | --- | --- | --- | --- |
| **Role of the care manager** | Job prerequisites | Skills | - **Work in a multidisciplinary team** - Motivate - Have organizational autonomy - Be versatile (multi-skilled) - Support | **16**  10  8  8  8 | - Adapt to the severity of the pathology - Be rigorous - Comply with specifications - Organize follow-ups | 7  7  6  5 |
|  |  | Soft skills | - Adaptable /available - Involved in the patient's life - Be attentive | 9  7  5 | - Maintain a professional posture - Know your limits as a care manager - Empathetic | 5  4  3 |
|  |  | Knowledge | - Requires experience - Transversal knowledge | 7  6 | - Requires training - Be trained on the digital monitoring platform | 4  3 |
|  | Job objectives | Organization and follow-up | - **Relay/link** - **Patient referral coordinator** - Long-term follow-up | **21**  **15**  14 | - Administrative work (data) - Home follow-up - Work with the patient's entourage | 8  7  6 |
|  |  | Health status and behaviours | - **Support/advice** - Meeting patients' needs - Maintaining benefits and behaviours - Make an overall assessment - Promote patient compliance | **18**  13  11  6  5 | - Limit hospitalization - Make patient autonomous - Improve patient health - Provide patient education - Limit medical complications | 4  4  3  3  2 |
| **Care manager experience feelings** | On the job |  | - **Importance of the Care Manager profession** - **Satisfaction with approach** - Sense of pride - Time-consuming - Feeling powerless / helpless - Skills development - Loneliness in the role of care manager | **19**  **17**  14  12  12  11  11 | - Recommends the job of care manager - Innovative accompaniment - Approach to be developed in the future - Evolution of work - Good preparation/supervision of piloting - A landmark for patients | 10  8  8  7  6  2 |
|  | On follow-up |  | - Patient satisfaction with follow-up - Patient weariness/dropout - Changes in patient behaviour - Patients make follow-up their own | 12  11  8  7 | - Patients seeking follow-up (keep motivation) - Limits patient loneliness - Patient anxiety about stopping follow-up | 6  4  2 |
|  | On the patient relationship |  | - Better patient understanding - More personal relationship (non-medical) - Strong patient relationship - Relationship of trust | 12  10  8  4 | - Care manager present at every stage of the disease - Feeling of disturbing the patient - Challenge to create a link - Refractory patient | 3  3  2  2 |
| **Technical aspects of monitoring** | Impact of remote support |  | - Remote monitoring does not change the patient relationship - Relationships built in person | 9  8 | - Reach new patients (off-site) - Difficult transition from presential to remote support | 3  3 |
|  | Feedback on modalities |  | - Satisfaction with remote follow-up - Preference for video over telephone | 4  1 | - Longer video interviews than telephone interviews | 1 |
| **Difficulties encountered** | Technical |  | - Complicated use of video | 6 | - Numerical tool problem – Application | 3 |
|  | Organizational |  | - Unknown role / lack of recognition - Insufficient dedicated time - Difficulty enrolling patients in a new course - Difficulty for care manager to stop follow-up | 10  10  9  4 | - Follow-up not relevant for all patients - Logistics issues - Questioning larger-scale feasibility - Difficulties for follow-up in small structures | 4  3  2  1 |
| **Suggested improvements** | Course organization |  | - Upstream preparation of follow-up with stakeholders - Reinforcing the city-hospital link - Review the team's role allocation | 9  4  4 | - Change the care manager/patient pairing to revive the dynamic - Bring patient back for physical tests - Reinforce actions for patients at risk of dropping out/non-adherence | 3  2  2 |
|  | Individualize follow-up modalities |  | - **Personalize follow-up frequency/duration** - Personalize monitoring to suit each patient | **19**  11 | - Customize questionnaires / adapt them to the patient | 7 |

**S5: 15-point checklist of criteria for good thematic analysis**

| Process | No. | Criteria | Response |
| --- | --- | --- | --- |
| Transcription | 1 | The data have been transcribed to an appropriate level of detail, and the transcripts have been checked against the tapes for ‘accuracy’. | Yes. |
| Coding | 2 | Each data item has been given equal attention in the coding process. | Yes. The two coders have discussed and validated all codes. |
|  | 3 | Themes have not been generated frow few vivid examples (an anecdotal approach), but instead the coding process has been thorough, inclusive and comprehensive. | The coding process has been thorough, inclusive and comprehensive, enabling the generation of themes in accordance with the method. |
|  | 4 | All relevant extracts for all each theme have been collated. | Yes. |
|  | 5 | Themes have been checked against each other and back to the original data set. | Yes. |
|  | 6 | Themes are internally coherent, consistent, and distinctive. | Yes, the codes have been organised into distinct themes. |
| Analysis | 7 | Data have been analysed – interpreted, made sense of – rather than just paraphrased or described. | Yes, data have been interpreted and made sense of. |
|  | 8 | Analysis and data match each other – the extracts illustrate the analytic claims. | Yes, illustrative extracts have been reported in the results section. |
|  | 9 | Analysis tells a convincing and well-organized story about the data and topic. | Yes, the analysis provides coherent information on the topic and the research question. |
|  | 10 | A good balance between analytic narrative and illustrative extract is provided. | Yes, narrative analysis is illustrated using extracts from patients and care managers to maintain a balanced results section. |
| Overall | 11 | Enough time has been allocated to complete all phases of the analysis adequately, without rushing a phase or giving it a once-over-lightly. | Yes, dedicated time has been allocated to each phase of the analysis, with no time constraints. |
| Written report | 12 | The assumptions about, and specific approach to, thematic analysis are clearly explicated. | Yes, this is detailed in the methods section. |
|  | 13 | There is a good fit between what you claim you do, and what you show you have done – ie, described method and reported analysis are consistent. | Yes, the methods and results sections are consistent. |
|  | 14 | The language and concepts used in the report are consistent with the epistemological position of the analysis. | Yes. |
|  | 15 | The researcher is positioned as *active* in the research process; themes do not just ‘emerge’. | Yes, all themes were conceived through discussions between the two coders and validated by the project team. |

**S6: Standards for Reporting Qualitative Research (SRQR) checklist**

| **Title and abstract** | |
| --- | --- |
| **Title** - Concise description of the nature and topic of the study; identifies qualitative approach (e.g., ethnography, grounded theory, case study) or data collection methods (e.g., interview, focus group). | Y |
| **Abstract** - Summary of key elements of the study using the journal’s abstract format (typically background, purpose, methods, results, conclusions). | Y |
| **Introduction** | |
| **Problem formulation** - Description and significance of the problem/phenomenon studied; review of relevant theory and empirical work; problem statement. | Y |
| **Purpose or research question** - Purpose of the study and specific objectives or questions. | Y |
| **Methods** | |
| **Qualitative approach and research paradigm** - Qualitative approach (e.g., ethnography, grounded theory, case study, phenomenology, narrative research) and guiding theory/constructivist/interpretivist stance; rationale. | Y |
| **Researcher characteristics and reflexivity** - Researchers’ characteristics that may influence research (e.g., personal attributes, relationship with participants, assumptions); potential interactions with participants; reflexive positioning. | Y |
| **Context** - Setting/site and salient contextual factors; rationale. | Y |
| **Sampling strategy** - How and why participants/documents/events were selected; criteria for sampling; when sampling ceased (e.g., saturation); rationale. | Y |
| **Ethical issues pertaining to human subjects** - Documentation of approval by ethics board and participant consent; confidentiality/data security. | Y |
| **Data collection methods** - Types of data collected; details of procedures including start/end of collection; iterative processes; triangulation of sources/methods; rationale. | Y |
| **Data collection instruments and technologies** - Description of instruments (e.g., interview guides, questionnaires) and devices (e.g., audio recorders); how instruments changed over the course of the study. | Y |
| **Units of study** - Number and relevant characteristics of participants/documents/events included; level of participation (e.g., dropouts). | Y |
| **Data processing** - How data were processed (e.g., transcription), data management and security, verification/integrity checks, coding, anonymization/de-identification. | Y |
| **Data analysis** - Process by which inferences/themes were identified and developed; use of researchers; rationale. | Y |
| **Techniques to enhance trustworthiness** - Techniques to enhance credibility (e.g., member checking, audit trail, triangulation); rationale. | Y |
| **Results/findings** | |
| **Synthesis and interpretation** - Main findings (e.g., interpretations, inferences, themes); linkage to theory or model if applicable. | Y |
| **Links to empirical data** - Evidence (e.g., quotes, field notes, text excerpts, photographs) supporting analytic findings. | Y |
| **Discussion** | |
| **Integration with prior work, implications, transferability, and contribution(s) to the field** - Short summary of main findings; comparison with prior research; scope of application/generalizability; unique contribution(s). | Y |
| **Limitations** - Trustworthiness and limitations of findings. | Y |
| **Other** | |
| **Conflicts of interest** - Potential sources of influence or perceived influence; how managed. | Y |
| **Funding** - Sources of funding and other support; role of funders in data collection, interpretation, and reporting. | Y |

Legend: Y = addressed in manuscript; N = not addressed; NA = not applicable.
